# Correction: Noble metal supported hexagonal boron nitride for the oxygen reduction reaction: a DFT study

**DOI:** 10.1039/c8na90003e

**Published:** 2018-11-30

**Authors:** Seoin Back, Samira Siahrostami

**Affiliations:** SUNCAT Center for Interface Science and Catalysis, Department of Chemical Engineering, Stanford University Stanford CA 94305 USA; Department of Chemistry, University of Calgary 2500 University Drive NW Calgary Alberta Canada T2N 1N4

## Abstract

Correction for ‘Noble metal supported hexagonal boron nitride for the oxygen reduction reaction: a DFT study’ by Seoin Back *et al.*, *Nanoscale Adv.*, 2019, DOI: 10.1039/c8na00059j.

One of the affiliations of the corresponding author was omitted from the original manuscript. The correct affiliations are as shown above.

The Royal Society of Chemistry apologises for these errors and any consequent inconvenience to authors and readers.

## Supplementary Material

